# Pacemakers and AICDs in the Magnet; have we turned the corner?

**DOI:** 10.1186/1532-429X-18-S1-P136

**Published:** 2016-01-27

**Authors:** Huma Samar, June A Yamrozik, Ronald B Williams, Mark Doyle, Moneal Shah, Christopher Bonnet, Robert W Biederman

**Affiliations:** 1 Cardiology, Loma Linda Veterans Administration Hospital, Los Angeles, CA USA; 2 grid.413621.30000000404551168Cardiac MRI, Allegheny General Hospital, Pittsburgh, PA USA

## Background

MR imaging is infrequently performed on patients with conventional pacemakers/ICD's. Multiple studies have documented the safety of MRI scans in patients with implanted devices, yet the diagnostic clinical value of this approach has not been established not even considered.

### Hypothesis

We propose that MRI imaging patients with a pacemaker is crucial to the existing diagnosis and in many instances, substantially modifies diagnosis and patient management.

## Methods

An evaluation of 157 consecutive patients with PM/AICD's who underwent MRI (GE CV/i, 1.5T, GE, Milwaukee,WI) over 10 years (90% <5 years) was performed. All had PM's of which 12 were Bi-V PM's while 31 patients also had an AICD. Specific criteria were followed to objectively determine if the diagnosis via MRI enhanced patient care. Accordingly, four questions were answered following scan interpretation by both the MRI technologist and MRI physician(s):

1. Did the primary diagnosis change?
2. Did the MRI provide additional information to the existing diagnosis?
3. Was the pre-MRI (tentative) diagnosis confirmed?
4. Did patient management change?

If 'Yes' was answered to any of the above questions, it was considered that MRI was of value to patient diagnosis and/or impending therapy.

## Results

The average MRI scan time was 20 ± 55 minutes. Regarding the population, of the 157 patients imaged, 114 (73%) were neurology/neurosurgery cases, 7 (4%) were musculoskeletal and 36 (23%) were cardiac/vascular cases. After reviewing the 114 neurology/neurosurgery cases, 21 (18%) demonstrated MRI not only provided additional information but also changed the original diagnosis and in turn, the course of medical treatment. In 79 patients (69%) MRI provided additional, complementary diagnostic information. Thus, for 100 (88%), MRI scan was of value to the final diagnosis. In only 14 (12%) patients imaged did MRI not provide further information but simply confirmed the original diagnosis.

The 36 cardiac cases demonstrated that 5 patients (14%) the MRI provided additional information to change the original diagnosis and also patient management, 28 (77%) showed that complementary information was gathered while in 3(8%) the CMR was uninterpretable due to AICD artifact. In essence, 92% of the cardiac cases benefited by MRI performance.

Finally, in the 7 musculoskeletal cases, MRI provided additional information in 6 (85%) and in 1(15%), changed patient management.

Importantly, with careful attention to device reprogramming and scanner sequences, no safety issues were encountered and no adverse effects of undergoing the MRI scan were noted in any patient.

## Conclusions

MR imaging in patients with implanted pacemakers and defibrillators added substantial clinical value to patient diagnosis and subsequent management justifying the risk of the procedure. To our knowledge, this is the first study to focus *solely* on diagnostic value under the assumption that safety can be routinely accomplished.

